# ADAM9 contributes to vascular invasion in pancreatic ductal adenocarcinoma

**DOI:** 10.1002/1878-0261.12426

**Published:** 2019-01-09

**Authors:** Victor O. Oria, Paul Lopatta, Tatjana Schmitz, Bogdan‐Tiberius Preca, Alexander Nyström, Catharina Conrad, Jörg W. Bartsch, Birte Kulemann, Jens Hoeppner, Jochen Maurer, Peter Bronsert, Oliver Schilling

**Affiliations:** ^1^ Institute of Molecular Medicine and Cell Research University of Freiburg Germany; ^2^ Spemann Graduate School of Biology and Medicine University of Freiburg Germany; ^3^ Faculty of Biology University of Freiburg Germany; ^4^ Department of Biomedicine University of Basel Switzerland; ^5^ Department of Dermatology Medical Faculty Medical Center – University of Freiburg Germany; ^6^ Department of Neurosurgery Philipps University Marburg Germany; ^7^ Department of Anesthesiology, Intensive Care, and Pain Medicine University of Münster Germany; ^8^ Department of General and Visceral Surgery Medical Center – University of Freiburg Germany; ^9^ Faculty of Medicine University of Freiburg Germany; ^10^ Comprehensive Cancer Center Freiburg Medical Center – University of Freiburg Germany; ^11^ Department of Gynecology University Clinic RWTH Aachen Germany; ^12^ Institute of Surgical Pathology Medical Center – University of Freiburg Germany; ^13^ German Cancer Consortium (DKTK) and Cancer Research Center (DKFZ) Heidelberg Germany; ^14^ Tumorbank Comprehensive Cancer Center Freiburg Medical Center – University of Freiburg Germany; ^15^ Centre for Biological Signaling Studies BIOSS University of Freiburg Germany

**Keywords:** ADAM9, adhesion, angiogenesis, heparin‐binding EGF‐like growth factor, migration

## Abstract

A disintegrin and a metalloprotease (ADAM)‐9 is a metzincin cell‐surface protease with strongly elevated expression in solid tumors, including pancreatic ductal adenocarcinoma (PDAC). In this study, we performed immunohistochemistry (IHC) of a tissue microarray (TMA) to examine the expression of ADAM9 in a cohort of >100 clinically annotated PDAC cases. We report that ADAM9 is prominently expressed by PDAC tumor cells, and increased ADAM9 expression levels correlate with poor tumor grading (*P* = 0.027) and the presence of vasculature invasion (*P* = 0.017). We employed gene expression silencing to generate a loss‐of‐function system for ADAM9 in two established PDAC cell lines. *In vitro* analysis showed that loss of ADAM9 does not impede cellular proliferation and invasiveness in basement membrane. However, ADAM9 plays a crucial role in mediating cell migration and adhesion to extracellular matrix substrates such as fibronectin, tenascin, and vitronectin. This effect appears to depend on its catalytic activity. In addition, ADAM9 facilitates anchorage‐independent growth. In AsPC1 cells, but not in MiaPaCa‐2 cells, we noted a pronounced yet heterogeneous impact of ADAM9 on the abundance of various integrins, a process that we characterized as post‐translational regulation. Sprout formation of human umbilical vein endothelial cells (HUVECs) is promoted by ADAM9, as examined by transfer of cancer cell conditioned medium; this finding further supports a pro‐angiogenic role of ADAM9 expressed by PDAC cancer cells. Immunoblotting analysis of cancer cell conditioned medium highlighted that ADAM9 regulates the levels of angiogenic factors, including shed heparin‐binding EGF‐like growth factor (HB‐EGF). Finally, we carried out orthotopic seeding of either wild‐type AsPC‐1 cells or AsPC‐1 cells with silenced ADAM9 expression into murine pancreas. In this *in vivo* setting, ADAM9 was also found to foster angiogenesis without an impact on tumor cell proliferation. In summary, our results characterize ADAM9 as an important regulator in PDAC tumor biology with a strong pro‐angiogenic impact.

**Abbreviations**
ADAM: a disintegrin and a metalloprotease
APP: amyloid precursor protein
ASM: active site mutation
ATCC: American Type Culture Collection
BCA: bicinchoninic acid assay
BrdU: bromodeoxyuridine
CCM: cell conditioned medium
CXCR2: C‐X‐C chemokine receptor type 2
DMEM: Dulbecco's modified Eagle's medium
ECGM: endothelial cell growth medium
ECM: extracellular matrix
EGFR: epidermal growth factor receptor
eNOS: endothelial nitric oxide synthase
ERK: extracellular signal‐regulated kinase
FACS: fluorescence‐activated cell sorting
FAK: focal adhesion kinase
FCS: fetal calf serum
FFPE: formalin‐fixed paraffin‐embedded
FRET: fluorescence resonance energy transfer
GFP: green fluorescent protein
H&E: hematoxylin and eosin
HB‐EGF: heparin‐binding EGF‐like growth factor
HGF: hepatocyte growth factor
HUVEC: human umbilical vein endothelial cell
IHC: immunohistochemistry
ITG: integrin
mAb: monoclonal antibody
MEK: mitogen‐activated protein kinase kinas
MMP: matrix metalloprotease
MT1‐MMP: membrane‐type matrix metalloproteinase 1.
PDAC: pancreatic ductal adenocarcinoma
pERK: phosphorylated ERK
pFAK: phosphorylated FAK
PI3K: phosphatidylinositol‐3‐kinase
pMEK: phosphorylated MEK
PrAMA: proteolytic activity matrix analysis
qPCR: quantitative PCR
sAPP: soluble APP
SD: standard deviation
shRNA: small hairpin RNA
TCGA: The Cancer Genome Atlas
TMA: tissue microarray
VCAM: vascular cell adhesion molecule
VEGF: vascular endothelial growth factor
wt: wild type

## 1 Introduction

Pancreatic ductal adenocarcinoma (PDAC) is the most common malignancy of the pancreas and the fourth leading cause of cancer deaths worldwide with a 5‐year survival rate of > 10% (Cid‐Arregui and Juarez, 2015; Siegel *et al*., 2017). Members of the ADAM proteases play a pivotal role in cancer progression by driving several processes including angiogenesis, adhesion, cell–cell and cell–matrix interactions, migration, proliferation, and tumor invasion (Amour *et al*., 2002; Duffy *et al*., 2011; Giebeler and Zigrino, 2016; Mullooly *et al*., 2016). ADAM9 is a proteolytically active member of the ADAM family of proteases and is described to be involved in various tumorigenic processes. It is upregulated in many solid tumors such as breast, brain, lung, gastric, skin, liver, and pancreas, and its expression levels often correlate with disease progression and a poor prognosis (Fan *et al*., 2016; Giebeler *et al*., 2017; Grutzmann *et al*., 2005; Kim *et al*., 2014; Kohga *et al*., 2010; Kossmann *et al*., 2017; O'Shea *et al*., 2003). Multiple studies report the regulation of ADAM9 expression in tumors by supposedly tumor‐suppressive micro‐RNAs (Hamada *et al*., 2012; Van Kampen *et al*., 2017; Yuan *et al*., 2017).

Mice lacking ADAM9 are viable and do not present an overt phenotype (Weskamp *et al*., 2002). However, the lack of ADAM9 prevents craniofacial abnormalities that occur in mice lacking the cell surface protease MT1‐MMP (Chan *et al*., 2012). Furthermore, it became evident that a lack of ADAM9 leads to retinal degeneration in mice and cone‐rod dystrophy in humans, respectively (Parry *et al*., 2009). A recent study demonstrates involvement of ADAM9 in chronic obstructive pulmonary disease (Wang *et al*., 2018). Functional studies focusing on the role of ADAM9 in tumor biology are scarce. In the murine HGF/CDK4 melanoma model, ADAM9 contributes to tumor formation and metastatic load (Giebeler *et al*., 2017). That study also highlighted a pivotal role of ADAM9 in trans‐endothelial migration, which was corroborated by other studies (English *et al*., 2017; Micocci *et al*., 2016). Interaction of tumor–cell ADAM9 with platelet α6β1 integrin fosters platelet activation and tumor cell extravasation (Mammadova‐Bach *et al*., 2016).

Cell surface levels of ADAM9 are regulated via cytosolic interaction with sorting nexins, which in turn has an effect on ADAM9 activity (Mygind *et al*., 2018a). ADAM9 has been found to shed various cell surface proteins. Its most prominent substrate is heparin‐binding EGF‐like growth factor (HB‐EGF) (Izumi *et al*., 1998). Further documented substrates of ADAM9 include FGFR2iiib, EGF, amphiregulin, VE‐cadherin, EphB4, CD40, VCAM‐1, and Tie‐2 (Chan *et al*., 2012; Guaiquil *et al*., 2009; Peduto, 2009). At the same time, non‐catalytic functions of ADAM9 are equally important. For example, interaction of ADAM9 with β1 integrin may prevent integrin endocytosis, thereby increasing integrin surface expression (Mygind *et al*., 2018b).

Multiple studies highlight an involvement of ADAM9 in angiogenesis and vascularization in various disease settings. In lung cancer cells, ADAM9 expression contributes to a pro‐angiogenic expression signature (Kossmann *et al*., 2017; Lin *et al*., 2017). Mice lacking ADAM9 are partially protected from pathological retinal neovascularization in assays using oxygen‐induced retinopathy or laser‐induced choroidal neovascularization (Guaiquil *et al*., 2009). In PDAC, ADAM9 expression is remarkably increased (Alldinger *et al*., 2005; Grutzmann *et al*., 2004; Yamada *et al*., 2007). We aimed to characterize the role of ADAM9 in PDAC tumor biology using both *in vitro* and *in vivo* approaches.

## 2 Materials and methods

### 2.1 PDAC patient samples

Formalin‐fixed paraffin‐embedded (FFPE) tissue specimens from PDAC patients were used to stain for ADAM9 following ethical approval from the local institution ethics committee. Due to the retrospective study design and the dismal prognosis of pancreatic ductal adenocarcinomas, written informed consent was not available from all included patients. The presented study was positively reviewed by the local ethics committee (Ref: 61/15: Proteomic expression pattern in pancreatic carcinomas and metastases; Ethics Commission, Albert Ludwig's University of Freiburg, Germany). The study methodologies conformed to the standards set by the Declaration of Helsinki. The samples consisted of tumor specimens from 103 patients all diagnosed with ductal adenocarcinoma of the pancreas. Tumor histology was evaluated by an independent pathologist and patient data are summarized in Table 1. Before inclusion, patient data were anonymized.

**Table 1:** Description of the clinical and pathological tumor characteristics of the 103 patients used in this study. Correlation between ADAM9 expression and different clinicopathological parameters in PDAC patients. High ADAM9 expression correlated with tumor grade and vascular invasion (*P* < 0.05). ADAM9 levels were classified as either low or high based on the intensity of immunohistochemical staining

Variable	Total	ADAM9 levels		*P* value	Mean survival (years)	*P* value (Mean survival)
		Low (%)	High (%)			
ADAM9 semiquantative	101	51	50			
< Median (low)	50				2.61	0.41
> Median (high)	51				2.25	
Grading	103					
Good and moderate differentiated	59	34.30%	23.50%	0.03	2.61	0.1
Poorly differentiated	44	15.70%	26.50%		1.72	
Tumor size (Diameter in cm)	100					
< 5	58	28.30%	29.30%	0.93	2.42	0.99
> 5	42	21.20%	21.20%		2.07	
Lymph node ratio	102					
< 0.09	50	27.70%	20.80%	0.19	3.42	< 0.01
> 0.09	52	22.80%	28.70%		1.33	
Distant metastasis	103					
M0	100	49.00%	48.00%	0.56	2.41	0.42
M1	3	1.00%	2.00%		1.34	
Lymphatic invasion	103					
Absent	45	25.50%	18.60%	0.17	2.84	0.03
Present	58	24.50%	31.40%		1.82	
Vascular invasion	103					
Absent	86	46.10%	37.30%	0.02	2.55	< 0.01
Present	17	3.90%	12.70%		1.25	

### 2.2 Immunohistochemistry

Immunohistochemical staining of ADAM9 (PA5‐25959, Thermo, Rockford, IL, USA), Factor VIII (Dako, Santa Clara, CA, USA, IR527), and Ki‐67 (Abcam, Cambridge, UK, ab833) was performed as described earlier (Kohler *et al*., 2015; Müller *et al*., 2017). Briefly, 2‐μm tissue sections were deparaffinized and subjected to heat‐induced antigen retrieval. Tissue sections were then stained using the following steps: incubation in H_2_O_2_ for 5 min, with primary antibodies for 1 h, with mouse/rabbit linker (15 min), with horseradish peroxidase and secondary antibody for 20 min, and final incubation with 3, 3′‐diaminobenzidine for 10 min. Sections were then counterstained in hematoxylin for 1 min, with xylene used as permanent mounting medium.

### 2.3 Cell culture

Human PDAC cell lines AsPC‐1 and MiaPaCa‐2 were obtained from the American Type Culture Collection (ATCC). AsPC‐1 cells were cultured using RPMI 1640 medium supplemented with 10% fetal calf serum (FCS) and 1% antibiotics (penicillin/streptomycin). MiaPaCa‐2 cells were cultured using Dulbecco's modified Eagle's medium (DMEM) supplemented with 10% FCS and 1% antibiotics (penicillin/streptomycin). Both cells were cultured at 37 °C in a humidified atmosphere containing 5% CO_2_.

### 2.4 Plasmids and viral transduction

ADAM9 knockdown was generated using the MISSION shRNA lentivirus system from Sigma‐Aldrich (St. Louis, MO, USA) according to the manufacturer's protocol. Three different shRNA constructs were used: shControl (non‐targeting shRNA), shRNA_1, and shRNA_2. Briefly, AsPC‐1 and MiaPaCa‐2 cells were seeded in 12‐well plates overnight, then infected with the three shRNA in culture medium supplemented with polybrene. Forty‐eight hours after transfection, both cell lines were selected using 1 μg·mL^−1^ puromycin for 2 weeks. ADAM9 knockdown efficiency was confirmed using qPCR and western blot as described above.

### 2.5 Determination of ADAM activities using proteolytic activity matrix analysis

For analysis of protease activities in cell supernatants, AsPC‐1 and MiaPaCa‐2 cells (shControl and shADAM9) were cultured to confluence on a six‐well plate and incubated in 1 mL serum‐free DMEM without phenol red. Following 24 h of incubation, cell conditioned medium (CCM) was collected, spun to remove debris, and subjected to the proteolytic activity matrix analysis (PrAMA) technique, as described previously (Conrad *et al*., 2017; Miller *et al*., 2011) using FRET‐based metalloprotease substrates with distinct ADAM selectivity profiles. Briefly, FRET substrates PEPDab005, PEPDab010, PEPDab013, and PEPDab014 (BioZyme Inc., Apex, NC, USA) were diluted to 10 μm in 50‐μL assay buffer (1 mm ZnCl_2_, 20 mm Tris‐HCl pH 8.0, 10 mm CaCl_2_, 150 mm NaCl, 0.0006% Brij‐35), and incubated with 50‐μL conditioned supernatants for 4 h at 37 °C on a 96‐well microtiter white opaque plate. Fluorescence units versus time were monitored in technical triplicates with a FLUOstar^®^ Optima (BMG Labtech, Ortenberg, Germany) using excitation and emission wavelengths of 485 and 530 nm, respectively. A non‐linear model was used for curve fitting as described by Miller *et al*. (2011), the signal of a negative control was subtracted (FRET‐substrate only), and time‐lapse fluorometric data were normalized to a positive control (0.01% trypsin). Protease activities were calculated with PrAMA by comparing the pattern of substrate cleavage rates for each sample with a matrix of known catalytic efficiencies for particular ADAM with 30% sampling error and threshold σ_T_ = 1.4 using matlab (2018a, MathWorks, Natick, MA, USA) (Conrad *et al*., 2017; Miller *et al*., 2011).

### 2.6 Quantitative PCR

ADAM9 expression in both cell lines was confirmed using quantitative PCR and western blot. For quantitative PCR, total RNA was isolated using the peQlab Total RNA kit followed by synthesis of cDNA. Quantitative PCR was performed using the primers shown below with the following cycling conditions: one cycle of 72 °C for 1 min; 40 cycles of 95 °C for 15 s, 58 °C for 30 s, 72 °C for 30 s; and one cycle of 72 °C for 5 min. The list of primers used are shown in Table 2.

**Table 2:** List of primers used in the qPCR experiment for RNA analysis

Target	Forward primer	Reverse primer
ADAM9	AATGATGGAAGAGGCGGAGG	TGAGGTCTGTTGAAAGCCTGG
β‐Actin	AGCACTGTGTTGGCGTACAG	CTCTTCCAGCCTTCCTTCCT
Integrin α2	ACTTTGTTGCTGGTGCTCCT	CGGATAGTGCCCTGATGACC
Integrin α6	AGCTGTGCTTGCTCTACCTG	CCGGGGTCTCCATATTTCCG
Integrin β1	GCCGCGCGGAAAAGATGAAT	CCACAATTTGGCCCTGCTTG
Integrin β4	AATGCAGCCGGTCTGACTC	GCCATCCTCTTCCTCCCTCT

### 2.7 Western blot

Protein extraction was done using radioimmunoprecipitation assay (RIPA) buffer supplemented with 10% Triton and protease inhibitor cocktail (EDTA, PMSF, and E64). Protein concentration was determined using the bicinchoninic acid assay (BCA) method and western blot performed under non‐reducing conditions (ADAM9 and β1 integrin) in 10% SDS/PAGE. All other blots were conducted under reducing conditions. The following antibodies were used: goat anti‐human ADAM9 (R&D AF939, 1 : 1000), rabbit anti‐human ERK1/2 (CST #4695, 1 : 1000), rabbit anti‐human pERK1/2 (CST #4370, 1 : 1000), rabbit anti‐human MEK1/2 (CST #9122, 1 : 1000), rabbit anti‐human pMEK1/2 (CST #9121, 1 : 1000), sheep anti‐human FAK (R&D AF4467, 1 : 200), rat anti‐human pFAK Y397 (R&D MAB4528, 1 : 500), rat anti‐human ITGA2 (R&D MAB12331, 1 : 500), rabbit anti‐human ITGA6 (CST #3750, 1 : 1000), mouse anti‐human ITGB1 (R&D MAB1778, 1 : 500), rabbit anti‐human ITGB4 (CST #4707, 1 : 1000), and rabbit anti‐human APP KPI domain (Millipore, Burlington, MA, USA, AB5302, 1 : 500). Mouse anti‐human GAPDH (Abcam ab8245: 1 : 5000) and mouse anti‐human tubulin (Sigma, St. Louis, MO, USA, T6199, 1 : 1000) were used as loading controls. The membranes were developed with the West Pico Chemiluminescent (Thermo‐Pierce, Rockford, IL, USA) substrate and peroxidase activity detected with a LumiImager device.

### 2.8 Cell proliferation

The effect of ADAM9 on cell proliferation was determined using the BrdU assay. Briefly, 100 μL of culture medium containing 2 × 10^3^ cells was added to a 96‐well plate and cultured for 48 h. A BrdU‐labeling solution of 10 μL per well was added followed by 24‐h incubation. The cells were then fixed at room temperature followed by addition of 100 μL per well anti‐BrdU‐POD solution and incubated for 90 min. The cells were washed three times and developed using 100 μL per well substrate solution. Sample absorbance in each well was measured at 370 nm with background measurement at 492 nm. The BrdU incorporation assay was confirmed using manual cell counting method.

### 2.9 Cell migration (gap closure assay)

Cell migration was investigated using the scratch assay method with 2‐well silicone inserts with a well‐defined and reproducible cell‐free gap. Before starting the migration assay, cells were serum‐starved for 18–24 h. After trypsinization and cell counting, 4 × 10^4^ cells were seeded per insert and cultured for 24 h. Afterwards, the inserts were gently removed, and the cells washed and sub‐cultured in fresh medium. The defined scratch areas were photographed at 0 and 24 h after removing the inserts. The area of the scratch was quantified using image j software (NIH, Bethesda, MD, USA).

### 2.10 Chemotactic and invasion assays

Chemotactic migration and invasion assays were conducted using the CytoSelect 24‐well colorimetric assay (Cell BioLabs Inc., San Diego, CA, USA, CBA‐100‐C) according to the manufacturer's protocol.

### 2.11 Extracellular matrix array for cell adhesion

Cell adhesion was done using a colorimetric extracellular matrix (ECM) Cell Adhesion Array Kit for different matrices in accordance with the manufacturer's instructions. The ECM‐coated wells were rehydrated with PBS for 10 min before starting the experiment. Following trypsinization, cells were resuspended in assay buffer and 1 × 10^5^ cells in 100 μL was added per well. The cells were incubated for 2 h to allow them to adhere to the matrices, followed by gentle washing to remove unbound cells. A staining solution of 100 μL per well was added followed by incubation at room temperature for 5 min. The plate was washed gently three times with deionized water before solubilizing the cell‐bound stain with 100 μL per well of the extraction buffer. Sample absorbance was measured at 570 nm on a microplate reader.

### 2.12 Anchorage‐independent growth

Anchorage‐independent growth was assayed using a protocol established by Gou *et al*. (2007) for the propagation of PDAC cells with stem cell properties. Briefly, 2% methylcellulose in DMEM F12 medium was mixed with 2X Gou medium to make 1× Gou medium. A 2‐mL aliquot of 1× Gou medium was placed in 2‐mL Eppendorf tubes, and 20 μL of cell suspension containing 2 × 10^4^ cells was added. The mixture was gently mixed by pipetting 15–20 times, and 100 μL of the cell suspension was added into each well of a low attachment 96‐well plate. Each cell line had 10 technical replicates per experiment. The plates were incubated under normal culture conditions for 7 days and the spheres then visualized under a microscope.

### 2.13 Surface integrin expression

Briefly cells were trypsinized, washed in PBS, counted, and 50 000 cells resuspended in 100 μL FACS buffer (PBS + 5 mm EDTA and 3% FCS). Alexa‐488 conjugated ITGA2 (FAB1233G, R&D, Minneapolis, MN, USA), ITGA6 (FAB1350G, R&D), ITGB1 (FAB17781G, R&D), and ITGB4 (FAB4060G, R&D) antibodies were added to the cell solution and incubated for 15 min in the dark at room temperature. Concentration‐matched mouse mAb IgG1 isotype control was used as a control. All antibodies were used in 1 : 100 dilutions. Cells were washed and resuspended in 200 μL FACS buffer followed by flow cytometry analysis. Results were further validated by western blots and quantitative PCR.

### 2.14 Chemosensitivity assay

Different studies have implicated members of the ADAM family (8, 9, 10, and 17) in chemotherapeutic resistance in ovarian, breast, colorectal, and glioblastoma tumors (Al‐Fakhri *et al*., 2003; Ebbing *et al*., 2016; Kyula *et al*., 2010; Ueno *et al*., 2018). To this end, we investigated the role of ADAM9 expression silencing in sensitizing AsPC‐1 and MiaPaCa‐2 cells to gemcitabine, the standard chemotherapy regimen for PDAC. Cells were treated with 1 μm gemcitabine for 72 h prior to protein extraction and quantitation of caspase‐3 activity as an indicator of apoptosis.

Caspase‐3 activity was measured according to the manufacturer's protocol (Enzo Life Science, Farmingdale, NY, USA). Briefly, 20 μg of whole cell protein lysates (maximum volume of 30 μL) was incubated with DEVD‐AMC (60 μm), a fluorogenic caspase‐3 substrate, to a final adjusted volume of 100 μL with caspase‐3 buffer (100 mm Hepes, pH 7.5, 10 mm DTT). Fluorescent activity was measured immediately at 37 °C every 2 min for 30 min (Tecan Infinite M200 microplate reader). Relative caspase‐3 activity was determined by calculating the slope of the linear regression.

### 2.15 Spheroid sprouting model

The effect of ADAM9 on angiogenesis was investigated using the endothelial cell sprouting model. Briefly, human umbilical vein endothelial cells (HUVECs) were cultured as monolayers in endothelial cell growth medium (ECGM) supplemented with 2% FCS. HUVEC were harvested and suspended in ECGM containing 20% methylcellulose and 2% FCS. A total of 625 cells were seeded in one hanging drop for assembly into one spheroid. After 24 h, the spheres were harvested and used to investigate sprout formation in a collagen matrix containing 1 : 1 rat tail type I collagen and 20% FCS‐methylcellulose. A 1‐mL aliquot of the collagen matrix containing 40 spheroids was added into a low attachment 24‐well plate and incubated for 30 min. Sprout formation was induced by adding 100 μL CCM (shControl, shRNA_1, and shRNA_2) grown in culture for 18–24 h. As a negative control, concentrated conditioned medium without any cells was used. The plate was incubated for 18–24 h followed by assessment of sprout formation microscopically. At least 25 spheroids per condition were photographed and sprout length assessed quantitatively using image j.

### 2.16 *In vivo* tumor mouse models

A mouse orthotopic model was established in 5‐week‐old BALB/c nude mice (Jackson Laboratory, Ellsworth, ME, USA) in accordance with institutional guidelines. Ketamine was used for anesthesia. The surgical area was sterilized with an iodine solution, and a small incision was made through the skin and abdominal wall. The spleen was gently pulled though the incision, exposing the pancreas. AsPC‐1 cells 2 × 10^6^ in 50 μL Matrigel solution were injected into the tail of the pancreas. The spleen and pancreas were gently replaced in the abdomen and the surgical site closed with 4–5 sutures. Six mice were used per condition (shControl, shRNA_1, and shRNA_2). The mice were monitored twice a week with bodyweight measured concurrently. The cell‐derived tumors were analyzed 28 days after implantation.

For subcutaneous mouse models, 5‐week‐old BALB/c nude mice (Jackson Laboratory) were used according to established institutional guidelines (Animal Care and Use Committee of the University of Freiburg, Germany). We subcutaneously injected 1 × 10^6^ AsPC‐1 cells in Matrigel® (BD Biosciences, Heidelberg, Germany) in both flanks of nude mice (*n* = 2 for control, *n* = 3 for shRNA_1, and *n* = 3 for shRNA_2). Tumors were measured twice a week with calipers for up to 6 weeks. At the end point (tumor diameter of 1 cm), mice were sacrificed and subcutaneous tumors excised. Maintenance of animal strains and work performed in both studies was carried out in accordance with institutional guidelines (Animal Care and Use Committee of the University of Freiburg, Germany) and the German law for animal protection (Tierschutzgesetz). The ethical approval registration number for this study is G16‐13.

### 2.17 Picrosirius red staining

To investigate changes in the tumor extracellular matrix organization, we opted for the Picrosirius red staining visualized under polarizing light to provide information on the thickness and parallel alignment of collagen fibrils in the tumors for both orthotopic and subcutaneous tumors. Picrosirius red staining was performed and visualized as previously described by Nyström *et al*. (2015). Briefly, the FFPE tissues were dewaxed and hydrated, and nuclei staining with Weigert's hematoxylin for 8 min and counterstaining with 0.1% Picrosirius red was carried out for 60 min. Tissue sections were imaged with an Axioplan 2 microscope (Zeiss, Oberkochen, Germany) under polarizing light using identical exposure time and settings. Quantitation of the staining was performed using image j after application of a minimal threshold for all images.

### 2.18 Hypoxia

For total hypoxia experiments, 200 000 cells per well were seeded into 6‐well plates and incubated for 24 h. The medium was replaced, plates were sealed in an Anaerocult^®^ A mini bag (Merck, Darmstadt, Germany) and incubated at 37 °C for 6, 24, and 48 h. To confirm the absence of oxygen, an oxygen sensing strip was added to each bag. Cell viability was confirmed using trypan blue assay. A 100‐μL aliquot of cell suspension was mixed with an equal amount of Trypan blue. The percentage of living cells was counted in duplicates, using an automated cell counter (Bio‐Rad, Hercules, CA, USA).

### 2.19 Statistical analysis

For all *in vitro* experiments, statistical analysis was done for at least three independent experiments with the two‐sided Student *t* test using graphpad prism 6.0 software (GraphPad Software, San Diego, CA, USA) with *P* > 0.05 considered significant.

## 3 Results

### 3.1 ADAM9 expression correlates to tumor grade and lymphangiogenesis in a cohort of PDAC specimens

**Figure 1:**
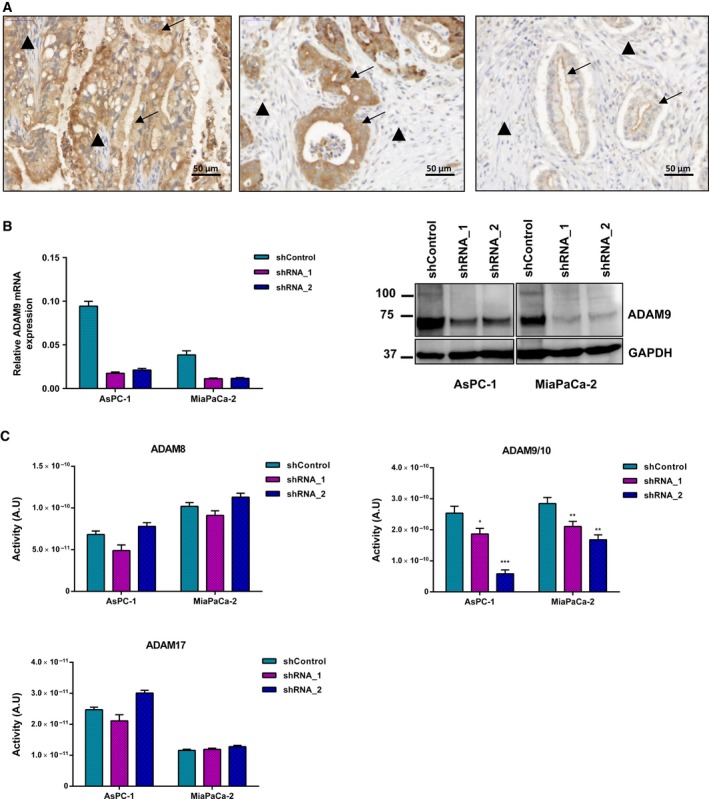
ADAM9 expression and activity in PDAC. (A) Representative immunohistochemical staining of ADAM9 in PDAC patients showing differential ADAM9 expression. ADAM9 expression is confined to the tumor cells (arrows) with limited or no expression in the stroma (arrowheads). Scale bar: 50 μm. (B) Lentiviral expression silencing of ADAM9 in AsPC‐1 and MiaPaCa‐2 cells using shRNA_1 and shRNA_2. (C) PrAMA inference analysis of CCM from AsPC‐1 and MiaPaCa‐2 (shControl versus shADAM9) using four fluorogenic substrates to detect ADAM activities. Results presented are absolute protease activities of the mean ± SD of three independent experiments. Statistical significance was determined using a two‐sided Student *t* test: *P < 0.05, **P < 0.01, ***P < 0.001.

As an initial step to investigate the involvement of ADAM9 in PDAC tumor biology, we investigated its expression in a cohort of >100 clinically annotated tumor samples by IHC analysis of a tissue microarray. An example of IHC staining is shown in Fig. 1A. Patient characteristics are summarized in Table 1. ADAM9 expression was predominantly observed in the tumor cells with limited or no expression in the stromal regions. ADAM9 expression was analyzed in a bivariate manner and correlated to patient characteristics in Table 1 **.** High ADAM9 expression in this patient cohort significantly correlated to advanced tumor grade (*P* = 0.027) and vascular invasion (*P* = 0.017). In this cohort, there was no significant correlation to tumor size, lymph node metastasis, distant metastasis, lymphatic invasion or overall survival (*P* > 0.1 in each case). According to German guidelines, PDAC patients with distant metastasis (M1) are ineligible for surgery (Dhayat *et al*., 2017). This justifies the lack of correlation between distant metastasis and overall patient survival in this case study as only three patients were (post‐surgically) found to have metastases. This number is too small to reach any statistical meaning.

In The Cancer Genome Atlas (TCGA) PDAC cohort, a strong association between ADAM9 expression and overall survival has been reported (Uhlen *et al*., 2017). Likewise, Grutzmann *et al*. (2004) report strong expression of ADAM9 in PDAC and association with shortened survival. Our study does not indicate such a correlation. There is no obvious explanation for these different findings. A possible bias may stem from clinical practice in the management of PDAC, including parameters such as selection of patients that are deemed eligible for surgery. The aforementioned studies did not investigate correlation of ADAM9 with lymphangiogenesis. However, as outlined in the Introduction, the observed association of ADAM9 expression with angiogenesis is in line with other reports, including reports on non‐tumor systems (Guaiquil *et al*., 2009; Parry *et al*., 2009).

### 3.2 ADAM9 expression silencing

To characterize functionally the role of ADAM9 in pancreatic cancer, we generated a stable loss of function systems in two established PDAC cell lines, AsPC‐1 and MiaPaCa‐2. Both cell lines display strong expression of ADAM‐9, which was markedly silenced upon stable transduction with either one of two different shRNA expression constructs: shRNA‐1 (Sigma, TRCN000046980) or shRNA‐2 (Sigma, TRC0000290529) (Fig. 1B).

Following ADAM9 expression silencing, we employed proteolytic activity matrix assay (PrAMA) for the detection of ADAM8, ADAM9, ADAM10, and ADAM17 activities in CCM. This method infers signals from a panel of moderately specific FRET polypeptide protease substrates to deduce a profile of specific ADAM proteolytic activities (Conrad *et al*., 2017; Miller *et al*., 2011). PrAMA analysis of specific ADAMs proteolytic activity showed no effect on ADAM8 and ADAM17 upon ADAM9 expression silencing (Fig. 1C). However, silencing of ADAM9 expression leads to a marked loss of activity for a shared ADAM9/10 substrate, which we attribute to the absence of ADAM9 (Fig. 1C).

### 3.3 ADAM9 does not contribute to cell proliferation or basement membrane invasion but facilitates migration

**Figure 2:**
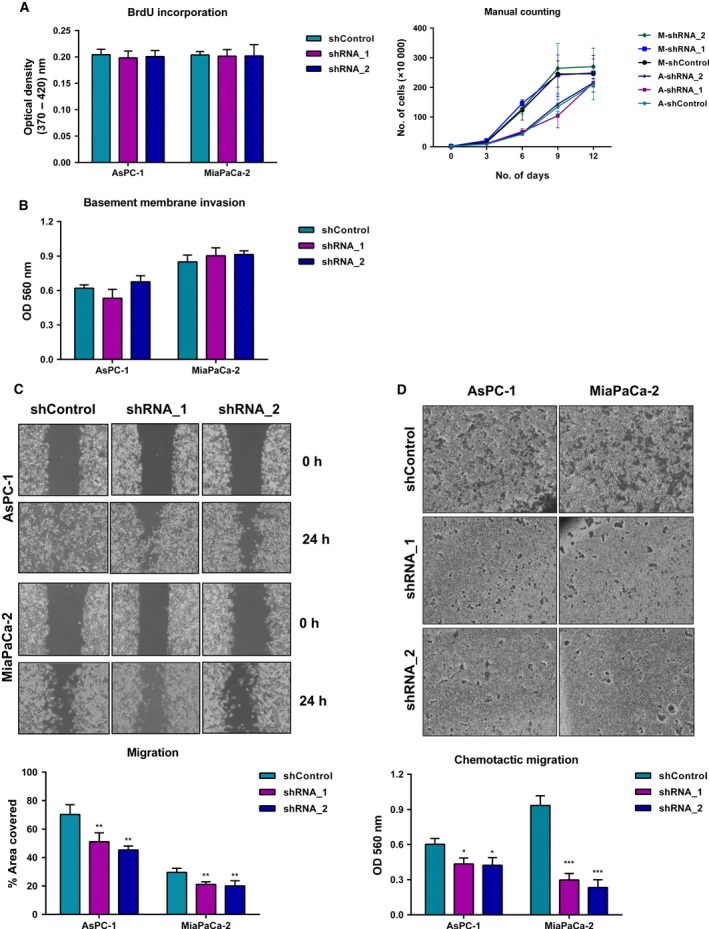
ADAM9 enhances migratory potential of PDAC cells. (A) ADAM9 expression has no effect on cell proliferation, either in the short or long term, as determined by BrdU assay and manual cell counting, respectively. (B) The invasive behavior assessed using the Transwell basement membrane invasion assay shows that ADAM9 expression silencing has no effect on the invasive behavior of PDAC cells. ADAM9 expression silencing impacts cell migration of both AsPC‐1 and MiaPaCa‐2 cells as determined by the gap closure method (C) and Transwell chemotactic migration assay (D). Data are expressed as the mean ± SD of three independent experiments. Statistical significance was determined using a two‐sided Student *t* test: *P < 0.05, **P < 0.01, ***P < 0.001.

The effect of ADAM9 on cell proliferation was determined using the BrdU incorporation assay and manual cell counting. In both assays, ADAM9 silencing did not affect cell proliferation (Fig. 2A). As an active protease, ADAM9 has been implicated in the processing of extracellular matrix components, which may facilitate tumor cell invasion (Sarkar *et al*., 2015; Zigrino *et al*., 2011). We investigated the role of ADAM9 in tumor cell invasion using a basement membrane invasion colorimetric assay (CBA‐100‐C). This assay involves probing cell invasion across an even‐layered dried basement membrane matrix that prevents discrimination between invasive and non‐invasive cells. ADAM9 expression silencing had no effect on cell invasion in either cell line (Fig. 2B).

Next, we investigated the role of ADAM9 in the motility of PDAC cells *in vitro,* using both the gap closure and chemotactic migration assay. Whereas AsPC‐1 and MiaPaca‐2 PDAC cell lines displayed differential motility profiles, silencing ADAM9 expression unanimously attenuated cell migration in both cell lines (Fig. 2C). The role of ADAM9 in chemotactic migration was investigated using the Transwell cell migration assay. Colorimetric analysis of the migrated cells showed that ADAM9 expression silencing reduced the ability of cells to migrate across the polycarbonate membrane in both ASPC‐1 and MiaPaCa‐2 cells (Fig. 2D). Our findings implicate ADAM9 in tumor cell migration. However, in ovarian clear cell carcinoma cells, ADAM9 was found to rather impede rather than foster cell migration, possibly suggesting cell‐type specificity of ADAM9 involvement in cell migration (Ueno *et al*., 2018).

### 3.4 ADAM9 mediates PDAC cell adhesion and anchorage‐independent growth *in vitro*

**Figure 3:**
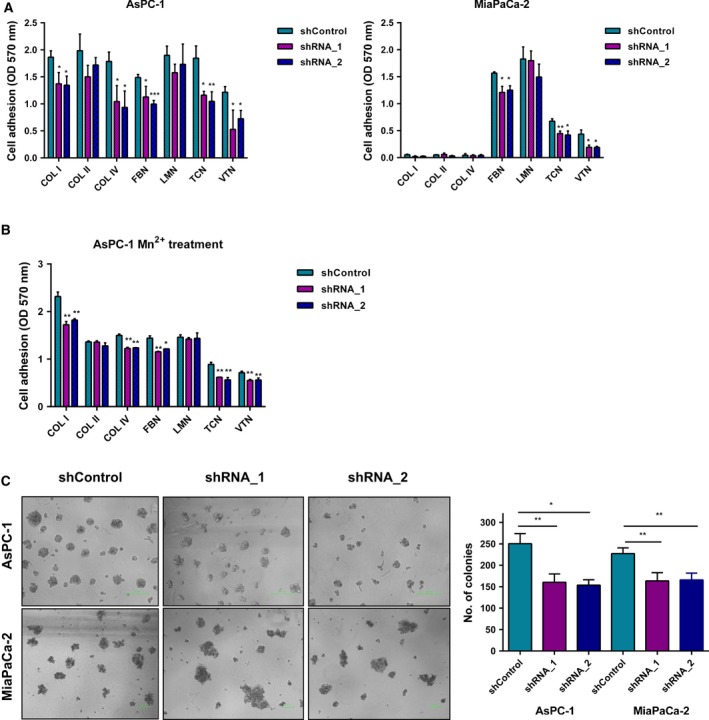
ADAM9 expression silencing reduces cell adhesion and anchorage‐independent growth. (A) Cells were plated on different matrices for 60 min, and cell adhesion was quantified colorimetrically at 570 nm. Adhesion to uncoated cell culture plasticware in the presence of BSA was used as control and subtracted from the raw optical density values. (B) Before plating, AsPC‐1 cells were treated with 1 mm Mn^2+^ for 5–10 min. Cells were then plated on different matrices for 60 min and adhesion determined as in (B). Integrin activation by Mn^2+^ did not rescue the adhesion phenotype. (C) Cells were grown in 1% methylcellulose on low‐attachment 96‐well plates for 7 days. The number of colonies per well were then manually counted and recorded. Data are expressed as the mean ± SD of three independent experiments. Statistical significance was determined using a two‐sided Student *t* test: *P < 0.05, **P < 0.01, ***P < 0.001.

Various reports point toward an impact of ADAM9 on cell adhesion (Cominetti *et al*., 2009; Mygind *et al*., 2018b). Generally, (tumor) cell–matrix interaction is a crucial aspect of tumor biology, including in PDAC. We chose to probe the impact of ADAM9 on PDAC tumor cell adhesion to a panel of ECM substrates. In both ASPC‐1 and MiaPaCa‐2 cells, adhesion to fibronectin, tenascin, and vitronectin was impaired upon reduced ADAM9 expression (Fig. 3A). In AsPC‐1 cells, ADAM9 expression silencing further impaired adhesion to collagens I and IV (Fig. 3A). Integrin activation by Mn^2+^ treatment failed to reverse this behavior (Fig. 3B), suggesting an integrin‐independent impact of ADAM9 on adhesion to these ECM substrates. Our results indicate a role of ADAM9 in mediating cell–matrix interactions and corroborate findings from earlier studies (Cominetti *et al*., 2009; Giebeler *et al*., 2017; Mygind *et al*., 2018b; Zigrino *et al*., 2011).

Anchorage‐independent growth is considered a hallmark of tumor malignancy, as it contributes to the ability of tumor cells to expand, invade adjacent tissues, and disseminate to distant organs, giving rise to metastasis (Guadamillas *et al*., 2011; Mori *et al*., 2009). To determine the role of ADAM9 in anchorage‐independent growth, we plated PDAC cells in 1% methylcellulose using low attachment plates. In both ASPC‐1 and MiaPaCa‐2 cells, silencing of ADAM9 expression significantly reduced the number of colonies formed (Fig. 3C), illustrating a pivotal role of ADAM9 in anchorage‐independent growth without an impact on actual cell proliferation.

### 3.5 ADAM9 impacts integrin abundance in a cell line‐dependent manner

**Figure 4:**
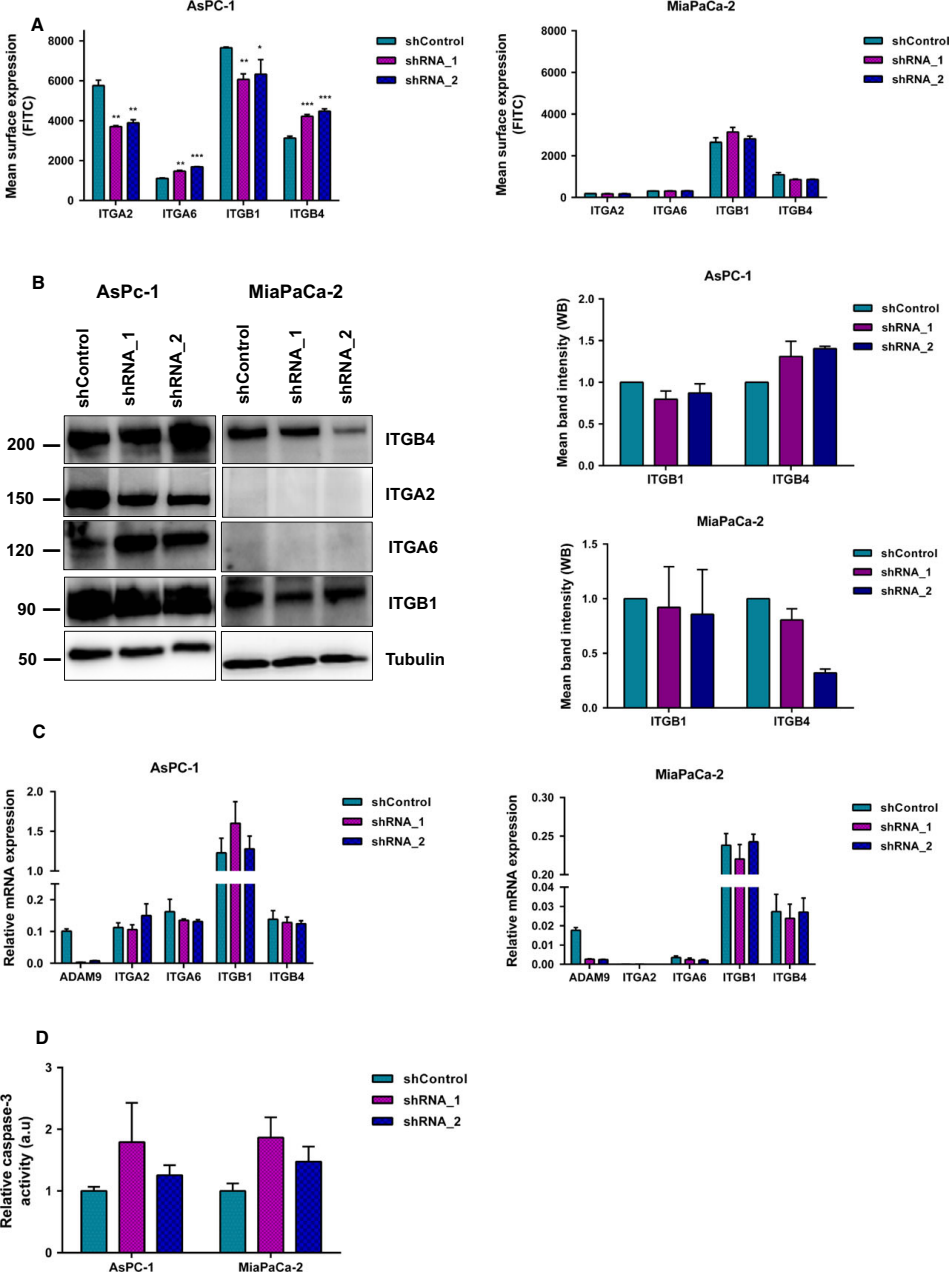
ADAM9 silencing impacts integrin abundance. (A) Surface expression analysis of ITGA2, ITGA6, ITGB1, and ITGB4 by flow cytometry using Alexa‐488 conjugated antibodies in both AsPC‐1 and MiaPaCa‐2 cells. (B) Western blot analysis of the impacted integrins in both AsPC‐1 and MiaPaCa‐2 cells upon ADAM9 expression silencing. For ITGB1 and ITGB4, we quantified the western blot band intensity to quantify changes at the protein level. We observed a slight decrease in ITGB1 and a slight increase in ITGB4 in AsPC‐1 cells upon silencing of ADAM9 expression. (C) No changes in integrin mRNA expression levels were observed upon silencing of ADAM9 expression. (D) Caspase‐3 activity was determined by fluorogenic DEVDase assay of cells pre‐treated with 1 μm gemcitabine for 72 h. All results are expressed as the mean ± SD of three independent experiments. Statistical significance was determined using a two‐sided Student *t* test: *P < 0.05, **P < 0.01, ***P < 0.001.

ADAM9 has been shown to interact with several integrins, which in turns drives different tumorigenic processes including adhesion, migration, extravasation, and metastasis (Mammadova‐Bach *et al*., 2016; Mygind *et al*., 2018b; Zigrino *et al*., 2011). Using flow cytometry, we evaluated the surface abundance of integrins α2, α6, β1, and β4 in AsPC‐1 and MiaPaca‐2 cells upon ADAM9 silencing. We noticed that AsPC‐1 cells, as compared with MiaPaCa‐2 cells, display elevated integrin levels (Fig.  4A,B), especially in the case of integrins α2 and α6. ADAM9 expression silencing led to a heterogeneous impact with regard to both the two cell lines and the integrins under investigation (Fig.  4A,B). The most prominent effects were observed in AsPC‐1 cells, with silencing of ADAM9 expression leading to lowered cell surface levels of integrins α2 and β1 but increased cell surface levels of integrins α6 and β4. In MiaPaCa‐2 cells, we observed a slight decrease in the surface expression of integrin β4, which we validated by immunoblotting (Fig.  4A,B). To investigate whether the changes in integrin levels were a result of changes in their gene expression, we analyzed their mRNA levels by qPCR. In both cell lines, no evident changes in the transcript levels were observed (Fig. 4C). This suggests a post‐translational regulatory effect of ADAM9 on integrins, a finding that has been reported in previous studies (Al‐Fakhri *et al*., 2003; Mygind *et al*., 2018b). Nevertheless, a consensus picture regarding the regulatory role of ADAM9 in integrin biology fails to emerge, as this aspect remains diverse with regard both to the actual integrin and the cellular system.

### 3.6 ADAM9 only marginally affects gemcitabine sensitivity in PDAC cells

A number of reports implicate members of the ADAM family (8, 9, 10, and 17) in the chemotherapeutic resistance in different solid tumors (Al‐Fakhri *et al*., 2003; Ebbing *et al*., 2016; Kyula *et al*., 2010; Ueno *et al*., 2018). For instance, Ueno *et al*. (2018) showed that ADAM9 suppresses cisplatin‐induced apoptosis in ovarian cancer cell lines, a phenotype abrogated in ADAM9 knockdown cells. Josson *et al*. (2011) investigated the sensitivity of C4‐2 prostate cancer cells to a panel of chemotherapy drugs (doxorubicin, docetaxel, etoposide, cisplatin, and gemcitabine) upon ADAM9 silencing. The authors found that ADAM9 silencing sensitized C4‐2 cells to these chemotherapeutic drugs (Josson *et al*., 2011). We probed the effect of ADAM9 expression silencing on the sensitivity of PDAC cell lines to gemcitabine, a standard mode of treatment for pancreatic cancer. Cells were treated with 1 μm gemcitabine for 3 days, trypsinized, and proteins extracted to determine caspase‐3 activity. Using the caspase‐3 activity assay, we show that ADAM9 silencing has only a negligible effect on gemcitabine sensitivity in both cell lines (Fig. 4D), although marginally elevated (albeit not meeting our significance threshold) levels of caspase‐3 activation were found in both PDAC cell lines for both ADAM9 knock‐down conditions.

### 3.7 ADAM9 contributes to a pro‐angiogenic expression signature of tumor CCM

**Figure 5:**
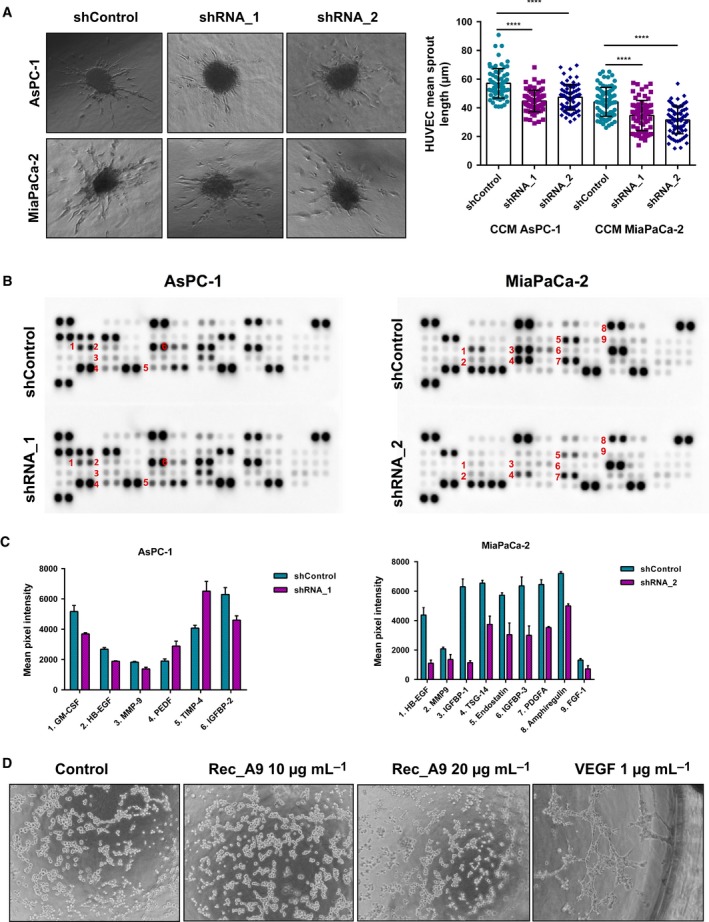
Effect of ADAM9 on angiogenesis. (A) HUVEC spheroids were treated with cell conditioned medium from shControl versus shADAM9 cells for 24 h followed by measurement of sprout lengths. At least 25 spheroids per condition were quantified from three independent experiments for each cell line. Results are expressed as the mean ± SD of three independent experiments. Statistical significance was determined using a two‐sided Student *t* test: ****P < 0.0001. (B) Human angiogenic array analysis was used to determine the release of pro‐angiogenic and anti‐angiogenic factors in AsPc‐1 and MiaPaCa‐2 cells. (C) Quantitation of the angiogenic array revealed six and nine differentially regulated factors in AsPC‐1 and MiaPaCa‐2 cells, respectively. (D) Incubation of HUVEC cells with recombinant ADAM9 only (10 and 20 μg·mL^−1^) did not promote tube formation, in contrast to VEGF (positive control, 1 μg·mL^−1^), where we observed tube‐like structures.

To address whether ADAM9 has an impact on angiogenesis *in vitro*, we exposed endothelial (HUVEC) cells to the culture medium conditioned by AsPC‐1 and MiaPaCa‐2 cells (shControl versus shADAM9). HUVEC spheroids exposed to conditioned medium from shControl cells displayed longer sprouts compared with spheroids exposed to conditioned medium from either AsPC‐1 or MiaPaCa‐2 cells with silenced ADAM9 expression (Fig. 5A). This supports findings from previous reports that emphasize a pro‐angiogenic function of ADAM9 in cancer (Guaiquil *et al*., 2009; Kossmann *et al*., 2017; Lin *et al*., 2017; Peduto, 2009). Moreover, using a medium‐transfer approach, these findings suggest that ADAM9 may be involved in the shedding and/or processing of pro‐angiogenic factors that drive sprout formation, a characteristic of angiogenesis.

To define a potential mechanism by which ADAM9 drives angiogenesis, conditioned media from shControl and shADAM9 cells were analyzed using an immunoblot array that probes the levels of a multitude of pro‐angiogenic and anti‐angiogenic proteins. As shown in Fig. 5B, in both AsPC‐1 and MiaPaCa‐2 cells, ADAM9 silencing markedly reduced the levels of soluble HB‐EGF, this being one of its annotated substrates. Further differences remained cell type‐specific. HB‐EGF has been previously described to promote angiogenesis in endothelial cells via the PI3K, ERK1/2, and eNOS signaling axis, but independently of vascular endothelial growth factor (VEGF) signaling (Mehta and Besner, 2007). These results suggest that tumor‐cell ADAM9 exerts a pro‐angiogenic function via the shedding of HB‐EGF. Addition of recombinant, soluble ADAM9 to serum‐free endothelial CCM did not promote tube formation (Fig. 5D), further substantiating the mediation of its impact by ectodomain shedding rather than ‘direct’ interaction of soluble ADAM9 with HUVEC cells.

### 3.8 ADAM9 affects auto‐/paracrine MEK/ERK signaling

**Figure 6:**
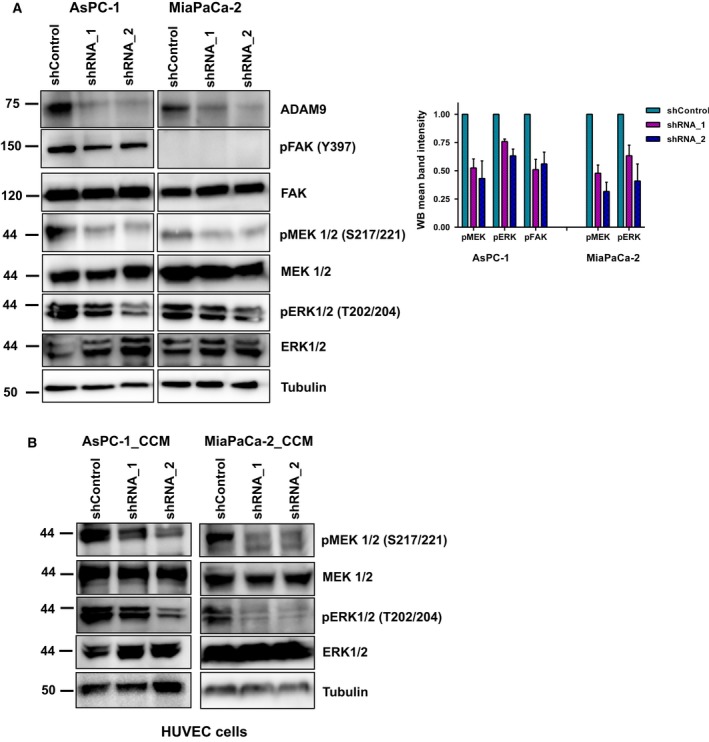
ADAM9 affects auto‐/paracrine MEK/ERK signaling. (A) Western blot of phosphorylation of MEK1/2 (S217/221) and ERK1/2 (T202/204) in AsPC‐1 and MiaPaCa‐2 cells following ADAM9 expression silencing. In AsPC‐1 cells, we observed decreased pFAK (Y397), which was absent in MiaPaCa‐2 cells. Quantitation of pFAK, pMEK1/2, and pERK1/2 in AsPc‐1 and MiaPaCa‐2 cells from three independent experiments. (B) Western blot analysis shows decreased phosphorylation of MEK1/2 (S217/221) and ERK1/2 (T202/204) in HUVEC cells following 30 min of incubation with cancer cell conditioned medium from both AsPC‐1 and MiaPaCa‐2 cells.

As a sheddase of epidermal growth factor receptor (EGFR) substrates, loss of ADAM9 expression likely affects MEK/ERK signaling in an autocrine or paracrine manner. Immunoblot analysis showed decreased phosphorylation of pMEK1/2 (S217/221) and pERK1/2 (T202/204) upon ADAM9 silencing (Fig. 6A). In AsPC‐1 cells, we additionally observed a decrease in the focal adhesion kinase (pFAK‐Y397) phosphorylation, which may be triggered by the differential integrin abundance in this cell line upon ADAM9 expression silencing. The decreased pMEK1/2 and pERK1/2 phosphorylation likely corresponds to decreased shedding of HB‐EGF and possibly amphiregulin, as observed in MiaPaCa‐2 cells (Fig. 5C).

To further support the paracrine HB‐EGF/EGFR signaling axis in endothelial cells, we incubated HUVEC cells in CCM from PDAC tumor cells (shControl and shADAM9) for 15 min. Immunoblot analysis of pMEK and pERK showed decreased phosphorylation in HUVEC cells incubated in shADAM9 conditioned medium from both cell lines (Fig. 6B). These results strengthen our hypothesis of HB‐EGF‐dependent angiogenesis signaling in the endothelial cells. It is well established that hypoxia increases HB‐EGF expression (Jin *et al*., 2002; Miyata *et al*., 2012; Munk *et al*., 2012). We probed ADAM9 levels under hypoxia in both AsPC‐1 and MiaPaCa‐2 cells at 6, 24, and 48 h (Fig. S1). In ‘early’ (up to 24 h) hypoxia, ADAM9 levels remained constant (AsPC‐1 cells) or even increased (MiaPaCa‐2 cells).

### 3.9 ADAM9 drives angiogenesis in murine pancreatic tumors

**Figure 7:**
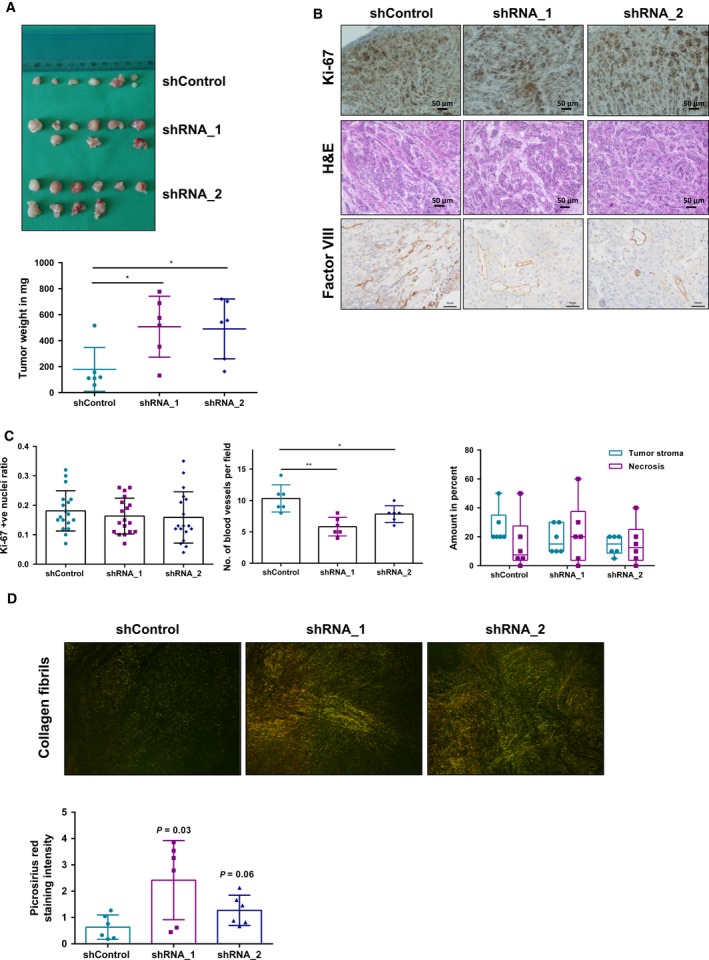
ADAM9 drives angiogenesis *in vivo*. AsPC‐1 cells were injected in nude mice and tumor growth monitored for 28 days. (A) Macroscopic images of isolated tumors from nude mice. Tumors from shADAM9 cells are heavier than shControl cells. (B) Immunohistochemical staining showing Ki‐67 and factor VIII expression in orthotopic tumors. H&E staining showing the tumor stroma ratio as well as necrotic areas in these orthotopic tumors. (C) Quantitation of Ki‐67 revealed no differences in tumor cell proliferation among the three mice groups. Three representative areas per tumor were selected and Ki‐67 was quantified using image j. Factor VIII staining of blood vessels was done to assess the extent of vascularization. Quantitation was done by averaging the number of blood vessels from three different regions per tumor. H&E staining of the orthotopic tumors revealed no differences in the average stromal component of these tumors or in the levels of necrosis. (D) Visualization of collagen fibers following Picrosirius red staining. Thick collagen fibers appear as orange‐red and thin fibers appear as green. Our results show that shADAM9 tumors have a higher density of thicker collagen fibers compared with shControl tumors: shRNA_1 (*P* = 0.03) and shRNA_2 (*P* = 0.06). Statistical significance was determined using a two‐sided Student *t* test: *P < 0.05, **P < 0.01. Scale bars: 50 μm.

To investigate the role of ADAM9 in pancreatic cancer *in vivo*, we chose an orthotopic murine model. Orthotopic injections of AsPC‐1 cells (shControl versus shADAM9) into the pancreas of nude mice were given (*n* = 6 for each condition). Tumors were grown for 28 days, followed by tumor harvesting. At the end point, pancreatic tumors formed from shADAM9 cells were larger in size compared with those from shControl cells (Fig. 7A). However, there was no difference in the number of proliferating cells as quantified by Ki‐67 staining (Fig. 7B,C). This result corroborates our *in vitro* data showing that ADAM9 does not affect cell proliferation. Tumor vascularization in the orthotopic pancreatic tumors was probed by factor VIII staining. Tumors grown from PDAC cells with wild‐type ADAM9 levels (shControl) displayed a higher number of blood vessels (normalized per tumor area) than did tumors grown from shADAM9 PDAC cells (Fig. 7B,C). This result is in line with our *in vitro* data on HUVEC sprout formation as well as with the association of ADAM9 expression with vascularization in our PDAC patient cohort. Taken together, our results emphasize a central role of ADAM9 in mediating tumor angiogenesis.

To explain the differences in tumor size, we used H&E staining to quantify the stromal component of these tumors. An analysis of the stromal area of these tumors suggests no significant differences in the levels of stromal infiltration. Moreover, manual analysis of necrotic regions using H&E staining revealed no differences in the levels of necrosis between the groups of mice (Fig. 7C).

**Figure 8:**
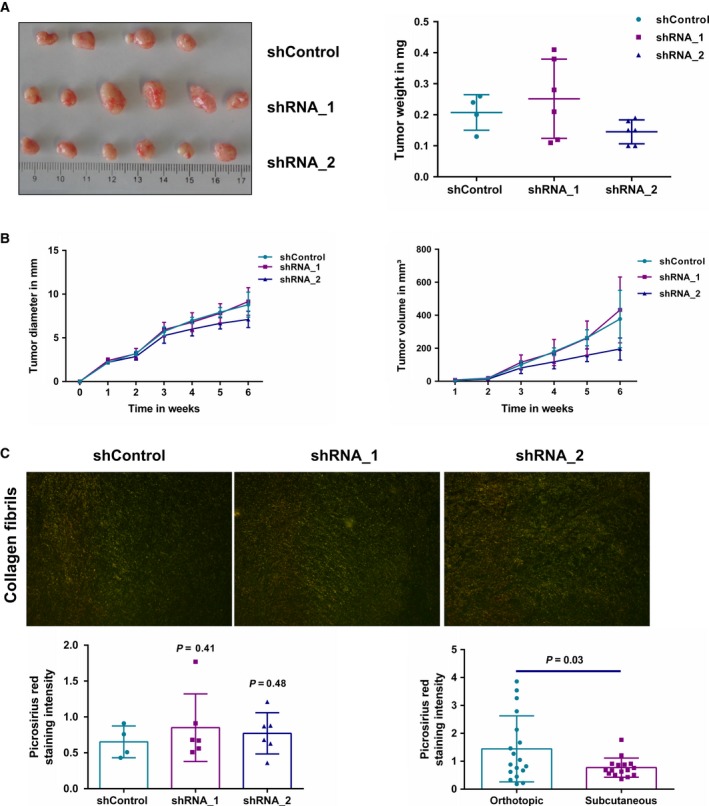
Subcutaneous mouse model. (A) Macroscopic images of subcutaneous isolated from nude mice after 6 weeks showed no differences in end point tumor weight. (B) Analysis of weekly tumor diameter and volume showed no differences between the mice groups. (C) Visualization of collagen fibers following Picrosirius red staining. Thick collagen fibrils appear as orange red and thin fibrils appear as green. No difference was observed in the collagen fiber arrangement and appearance between shControl and shADAM9 tumors (*P* > 0.05). However, the collagen density in the subcutaneous tumors was significantly lower than in orthotopic tumors (*P* = 0.03). Statistical significance was determined using a two‐sided Student *t* test.

To further investigate the ADAM9‐dependent difference in tumor volume, we resorted to a second tumor model. AsPC‐1 cells (shControl, shRNA_1, and shRNA_2) were injected subcutaneously into either flank of nude mice (*n* = 2 for shControl, *n* = 3 for each shRNA). Mice were monitored twice a week and tumors were harvested at the end point (1 cm diameter tumor size). An analysis of the end point tumor weight, tumor diameter, and tumor volume revealed no significant differences between the groups of mice (Fig.  8A,B, Table S1).

The subcutaneous tumor growth mimics our clinical data regarding ADAM9 not being associated with tumor size and growth. However, our orthotopic mouse model suggests larger tumors in the absence of ADAM9. We acknowledge this inconsistency between tumor growth/size in the orthotopic model and the clinical data. Nevertheless, it is important to note that despite the differences in tumor size in the orthotopic mouse model, there were no differences in Ki‐67 staining intensities between the shControl and shADAM9 groups. Based on these conflicting observations, we postulate that the ADAM9‐dependent difference in tumor volume is not an intrinsic property of the tumor cell but rather depends on the cellular/stromal microenvironment. For this reason, we investigated the remodeling of the extracellular matrix by quantifying the appearance and arrangement of fibrillar collagens in both tumor models. Our results showed no differences in the density of thicker, parallel collagen fibrils in the subcutaneous tumor model (Fig. 8C). However, in the orthotopic model, there was a pronounced tendency towards increased density of thickened parallel collagen fibrils in the shADAM9 tumors compared with in the shControl tumors (Fig. 7D). This increased density of thickened collagen fibrils in the shADAM9 tumors likely points to elevated ECM remodeling with a possible contribution to the observed tumor size differences. In addition, the orthotopic tumors displayed higher amounts of collagen fibril density compared with the subcutaneous tumors (Fig. 8C). Our work and further studies on ADAM9 generally point to some consensus findings on ADAM9 and multiple effects that remain specific for different types of model systems. An emerging consensus is a pro‐angiogenic impact of ADAM9. At the same time, the impact of ADAM9 on integrin biology or cell motility appears to vary between model systems. We consider the suppressing effect of ADAM9 on stromal remodeling in the orthotopic model to represent yet another example of a model‐specific effect.

### 3.10 Assessing catalytic and non‐catalytic functionality of ADAM9

**Figure 9:**
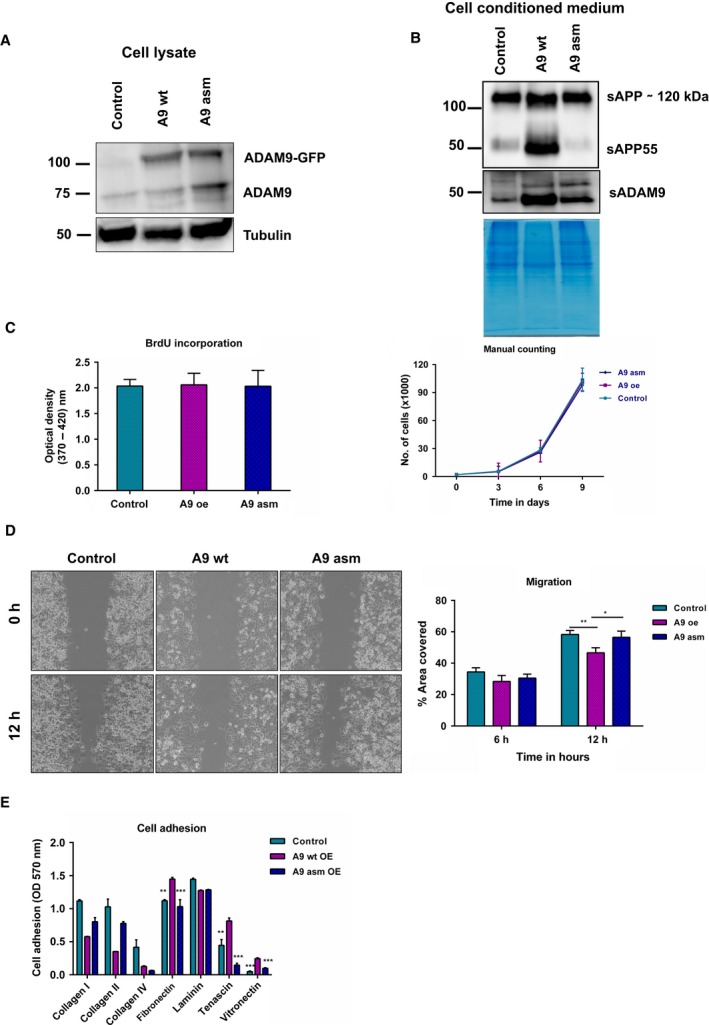
Catalytic and non‐catalytic functionality of ADAM9. (A) Immunoblot analysis of the overexpression of ADAM9 wild‐type (wt) and active site mutant E348A (asm) in Panc‐1 cells. (B) Detection of sAPP in cell conditioned medium of Panc‐1 cells. A processed fragment, sAPP55, was detected in cells overexpressing active ADAM9. A soluble fraction of ADAM9 was also detected in the cancer cell conditioned medium of these cells at around 50 kDa. (C) Overexpression of ADAM9 (wt and asm) has no effect on both short‐term or long‐term cell proliferation as determined by BrdU assay and manual cell counting. (D) Overexpression of ADAM9 (wt) attenuated cell migration in Panc‐1 cells as determined by the gap closure assay. (E) Expression of active ADAM9 (unlike inactive ADAM9) facilitated tumor cell adhesion to fibronectin, tenascin‐C, and vitronectin. All results are expressed as the mean ± SD of three independent experiments. Statistical significance was determined using a two‐sided Student *t* test: *P < 0.05, **P < 0.01, ***P < 0.001.

ADAM9 has been found to exert many of its biological roles through non‐catalytic functions such as protecting cell surface integrins from endocytosis and subsequent degradation (Mygind *et al*., 2018b). We noted that Panc‐1 cells express very low levels of endogenous ADAM9. We chose stably to overexpress GFP‐tagged human ADAM9 in Panc‐1 cells either with a wild‐type (wt) active site sequence or with an active site mutation (asm; mutated glutamic acid to alanine, E348A) (Fig. 9A). We focused on full length canonical ADAM9 rather than investigating constitutively spliced variants (Hotoda *et al*., 2002; Mazzocca *et al*., 2005). Autocatalytic processing has been implicated in the maturation and activation of some ADAM, resulting in the generation of soluble fractions (Blobel, 2005). A soluble fraction of ADAM9 (sADAM9) was detected in the CCM of Panc‐1 cells at about 50 kDa (Fig. 9B), an indication of autocatalytic shedding as previously reported for ADAM8 and 28 (Howard *et al*., 2000; Schlomann *et al*., 2002). The sADAM9 fraction was strongly elevated upon overexpression of GFP‐tagged human ADAM9 with a wild‐type active site sequence, demonstrating its proteolytic capacity. The catalytic activity of overexpressed GFP‐tagged ADAM9 was further illustrated by the shedding of amyloid precursor protein (APP). Immunoblot analysis (antibody directed against the Kunitz domain of sAPP) further revealed elevated levels of soluble APP (sAPP; ∼120 kDa) in Panc‐1 cells overexpressing GFP‐tagged human ADAM9 with a wild‐type active site sequence (Fig. 9B). This antibody also detected a band at approximately 50 kDa in the conditioned medium which was strongly enriched upon overexpression of GFP‐tagged human ADAM9 with a wild‐type active site sequence. Previous reports have demonstrated that the N‐terminal fragment of sAPP can be further processed by as yet unidentified proteases (Nikolaev *et al*., 2009; Sutinen *et al*., 2012). Generally, we consider the present results to support the proteolytic shedding capacity of overexpressed GFP‐tagged ADAM9. Overexpression of ADAM9, either catalytically active or inactive, did not affect cellular proliferation (Fig. 9C). Overexpression of active ADAM9 was found to impede migration of Panc‐1 cells, whereas no such effect was observed upon overexpression of catalytically inactive ADAM9 (Fig. 9D). Unlike AsPC‐1 and MiaPaCa‐2 cells, ADAM9 appears to counteract the motility of Panc‐1 cells, similar to the situation observed in ovarian carcinoma cells (Ueno *et al*., 2018). We also assessed whether adhesion of Panc‐1 cells was affected by the presence of active or catalytically inactive ADAM9. Expression of active ADAM9 (but not of catalytically inactive ADAM9) facilitated adhesion to fibronectin, vitronectin, and tenascin‐C (Fig. 9E). We consider this to constitute a rather unexpected finding for which the mechanistic underpinning remains beyond the scope of the present study.

## 4 Discussion

Our study highlights that ADAM9 plays an important role in PDAC tumor biology, affecting tumor angiogenesis, cell migration, adhesion to different ECM substrates, and anchorage‐independent growth. Our key finding, in line with other reports (Guaiquil *et al*., 2009; Kossmann *et al*., 2017; Lin *et al*., 2017), is the pro‐angiogenic role of ADAM9 both *in vitro* and *in vivo*. This angiogenic role of ADAM9 is dependent on HB‐EGF shedding in tumor cells that signal in a paracrine manner (MEK/ERK signaling) to endothelial cells. Kossmann *et al*. (2017) evaluated the critical role of ADAM9 in lung cancer metastatic process and angiogenesis *in vivo*. The authors showed that loss of ADAM9 led to interleukin 8 down‐regulation and interaction with its receptor CXCR2, thereby attenuating formation of neo‐vessels. Using a retinopathy mouse model, Guaiquil *et al*. (2009) demonstrated the importance of ADAM9 in angiogenesis. Their results showed that ADAM9 expression promotes pathologic retinal neovascularization by shedding pro‐angiogenic factors such as VE‐cadherin, vascular cell adhesion molecule (VCAM), CD40, Tie‐2, and EphB4 from endothelial cells (Guaiquil *et al*., 2009). These findings support the observed correlation between ADAM9 overexpression in PDAC patients and a vascular invasion phenotype, strongly suggesting a central role of ADAM9 in angiogenesis. Additional results also corroborate published findings that link ADAM9 to the pathogenesis and progression of different solid tumors (Fan *et al*., 2016; Giebeler *et al*., 2017; Grutzmann *et al*., 2005; Kim *et al*., 2014; Kohga *et al*., 2010; Kossmann *et al*., 2017; O'Shea *et al*., 2003). For instance, Mygind *et al*. (2018b) demonstrated the critical role of ADAM9 in cell migration and cell adhesion to collagen I and fibronectin. The authors showed that silencing ADAM9 expression lowered the focal adhesion kinase and cell adhesion machinery in part by affecting β1 integrin endocytosis and activity at the cell surface, which in turn reduced migration and adhesion.

A number of published studies have documented the central role of EGFR/MEK/ERK signaling in the development and progression of PDAC (Ardito *et al*., 2012; Diersch *et al*., 2016; Fitzgerald *et al*., 2015; Navas *et al*., 2012). Navas *et al*. (2012) showed that K‐Ras‐driven PDAC is essentially dependent on EGFR signaling. Using mouse models, they showed that the progression of acinar neoplasia to ductal metaplasia and finally to full‐blown PDAC required EGFR activation even in the presence of constitutively active K‐RAS^G12V^. This dependency on EGFR was further elaborated by Ardito *et al*. (2012) and Diersch *et al*. (2016) illustrating the key role of EGFR signaling in PDAC progression. Ardito *et al*. (2012) showed that oncogenic KRAS upregulates endogenous EGFR expression and activation driving PDAC development. Pharmacological inhibition or gene silencing of EGFR effectively eliminated KRAS‐driven PDAC development *in vivo*. Aberrant EGFR and ERK1/2 signaling are vital in tumor cell migration, invasion, and angiogenesis. As an active sheddase, ADAM9 is involved in the shedding of EGFR ligands such as HB‐EGF, amphiregulin, EGF, and epiregulin (Blobel, 2005). We observed ADAM9‐dependent shedding of HB‐EGF in both tumor cell lines as well as ADAM9‐dependent shedding of amphiregulin in MiaPaCa‐2 cells. Our results suggest an ADAM9‐dependent EGFR/MEK/ERK signaling pathway driven by EGFR ligands as previously described for ADAM10 and ADAM17 (Blobel, 2005). At the same time, our results emphasize that many aspects of ADAM9 functionality are strongly cell type and context‐dependent, e.g. with regard to cellular motility or its impact on cell surface integrin abundance. Likewise, an in‐depth distinction of catalytic and non‐catalytic functionality of ADAM9 remains an important topic for further investigation.

## 5 Conclusion

Using *in vitro* and *in vivo* systems, we demonstrated the role of ADAM9 in PDAC progression in driving crucial processes such as angiogenesis, cell migration, adhesion to different extracellular matrices, and anchorage‐independent growth. These processes are likely driven in part by ADAM9‐dependent shedding of HB‐EGF that signals via the EGFR/MEK/ERK axis.

## Conflict of interest

The authors declare no conflicts of interest.

## Authors' contributions

VOO performed the experiments and analyzed data. PL, TS, BTP, AN, and JM did the experiments. CC and JWB performed the PrAMA assay and data analysis. BK, JH and PB conceived the study and assembled the patient cohort, and OS conceived the study and analyzed data. All authors contributed to the writing of the manuscript.

## Supporting information


**Figure S1.** Analysis of AsPC‐1 (A,C) and MiaPaCa‐2 (B,D) cells grown under hypoxia. (A,B) Immunoblotting of ADAM9 with GAPDH used as a loading control. Coomassie staining of gel as additional loading control. Cell viability of cells under hypoxia was determined using the Trypan blue assay in (C) AsPC‐1 and (D) MiaPaCa‐2 cells.

**Table S1.** Calculation data of the volume of subcutaneous tumors.
