# Supplementary figures and images for: ADAM9 contributes to vascular invasion in pancreatic ductal adenocarcinoma
Source: Mol Oncol. 2019 Jan 9;13(2):456–79. doi: 10.1002/1878-0261.12426 (PMC6360373; doi:10.1002/1878-0261.12426)

Supplementary Figure 1

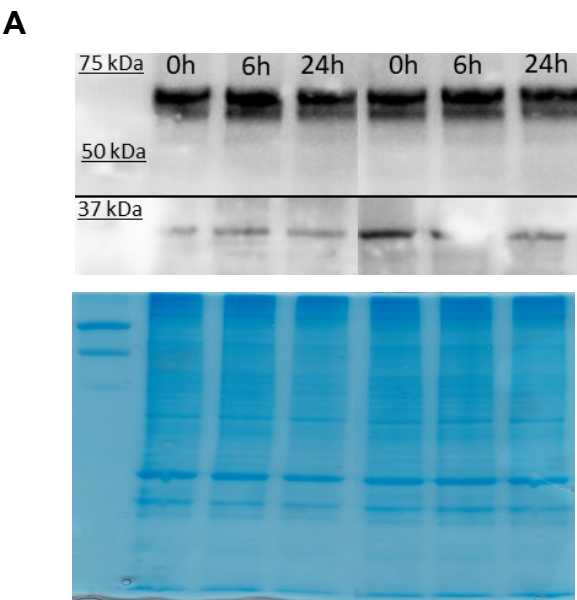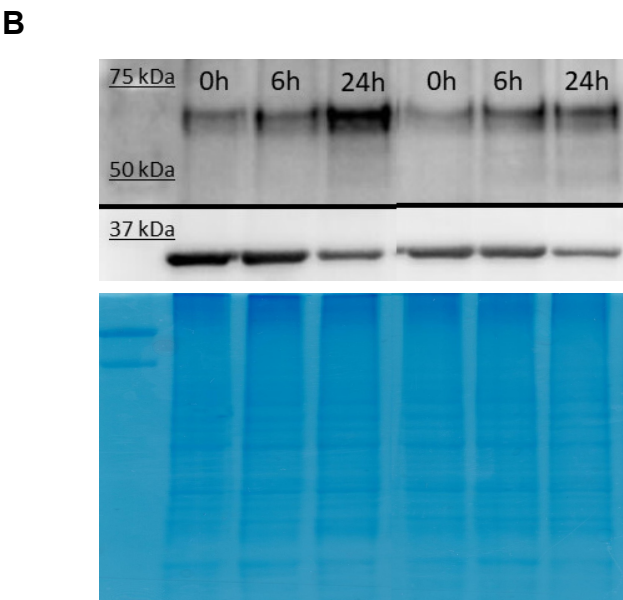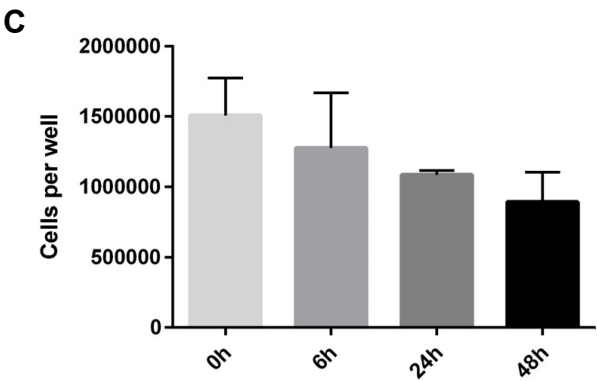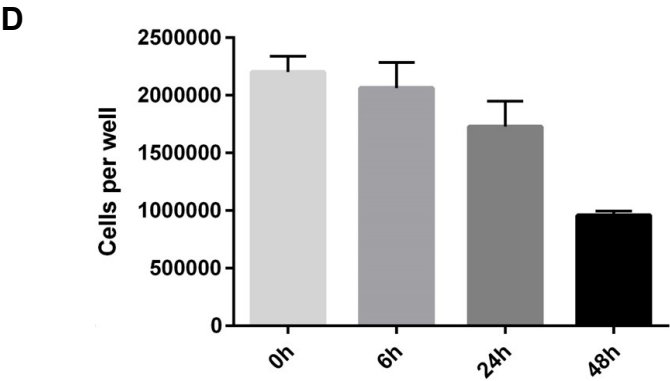

Supplement: Supplementary file 1 — Figure S1. Analysis of AsPC‐1 (A,C) and MiaPaCa‐2 (B,D) cells grown under hypoxia. (A,B) Immunoblotting of ADAM9 with GAPDH used as a loading control. Coomassie staining of gel as additional loading control. Cell viability of cells under hypoxia was determined using the Trypan blue assay in (C) AsPC‐1 and (D) MiaPaCa‐2 cells. [file MOL2-13-456-s001.pdf]
